# Accumulation of BDCA1^+^ Dendritic Cells in Interstitial Fibrotic Lung Diseases and Th2-High Asthma

**DOI:** 10.1371/journal.pone.0099084

**Published:** 2014-06-10

**Authors:** Alexandra M. Greer, Michael A. Matthay, Jasleen Kukreja, Nirav R. Bhakta, Christine P. Nguyen, Paul J. Wolters, Prescott G. Woodruff, John V. Fahy, Jeoung-Sook Shin

**Affiliations:** 1 Department of Microbiology and Immunology, Sandler Asthma Basic Research Center, University of California San Francisco, San Francisco, California, United States of America; 2 Department of Medicine and Anesthesia, University of California San Francisco, San Francisco, California, United States of America; 3 Division of Cardiothoracic Surgery, University of California San Francisco, San Francisco, California, United States of America; 4 Division of Pulmonary, Critical Care, Allergy and Sleep Medicine, University of California San Francisco, San Francisco, California, United States of America; University Hospital Freiburg, Germany

## Abstract

Dendritic cells (DCs) significantly contribute to the pathology of several mouse lung disease models. However, little is known of the contribution of DCs to human lung diseases. In this study, we examined infiltration with BDCA1^+^ DCs of human lungs in patients with interstitial lung diseases or asthma. Using flow cytometry, we found that these DCs increased by 5∼6 fold in the lungs of patients with idiopathic pulmonary fibrosis or hypersensitivity pneumonitis, which are both characterized by extensive fibrosis in parenchyma. The same DC subset also significantly increased in the lung parenchyma of patients with chronic obstructive pulmonary disease, although the degree of increase was relatively modest. By employing immunofluorescence microscopy using FcεRI and MHCII as the specific markers for BDCA1^+^ DCs, we found that the numbers of BDCA1^+^ DCs also significantly increased in the airway epithelium of Th2 inflammation-associated asthma. These findings suggest a potential contribution of BDCA1^+^ DCs in human lung diseases associated with interstitial fibrosis or Th2 airway inflammation.

## Introduction

Dendritic cells (DCs) play an important role in immune surveillance in the lungs. DCs located within or beneath the airway epithelia send projections toward the lumen to capture antigens in the airway [1]. DCs also reside in alveoli and capture antigens that have reached deep into the alveolar space [2]. Mice deficient in lung DCs exhibit significant defects in developing protective immunity against respiratory antigens [3], indicating that antigen capture and subsequent presentation by lung DCs plays an important role in inducing and propagating respiratory immunity. Interestingly, a number of studies suggest that DCs also play pathologic roles in several lung diseases, at least in mouse models. In a mouse model of viral lung infection, DCs produce chemoattractants that recruit Th2 cells that mediate persistent airway inflammation and mucus production [4]. DCs also both initiate and perpetuate Th2 inflammation driven by allergens in mice [5]. Furthermore, DCs contribute to persistent inflammation in murine lungs by mediating development and maintenance of tertiary lymphoid organs, which promote local activation of T cells [6]. However, relatively little is known about the contribution of DCs to human lung diseases.

Bronchial biopsies serve as an important source of accessible human lung tissue and immunohistochemistry is an important method to examine DCs in these small tissue samples. Previously, immunohistochemical analyses using antibodies to CD11c, DC-SIGN, CD1a, or Langerin have been performed to identify DCs in human bronchial biopsies [7]. These analyses have suggested that asthma, chronic obstructive pulmonary disease, and fibrosis are associated with a significant increase in DCs [7]–[10]. However, recent advances in characterization of human DC subsets reveal that many of these antibodies are neither specific nor sufficiently label broad human DC subsets. For example, CD11c is not only expressed in DCs but also highly expressed in monocytes and macrophages in humans [11]. DC-SIGN was found to be only expressed in monocyte-derived DCs, at least in mice [12]. CD1a and Langerin were found to be restricted to DC subsets specifically localized in the epithelium [13]. More recently, BDCA1 was found to be specifically expressed in the major DC subset in human blood [14]. Since this finding, anti-BDCA1 antibodies have been used to label DCs in various tissues, including lungs [13], [15]–[17]. Although this antibody labels a significant proportion of lung DC subsets, including CD1a or Langerin-expressing epithelial DCs [10], [18], [19], it additionally labels B cells [14]. Thus, immunohistochemistry-based methods of DC identification require an improved staining strategy by which DCs of a broadly representative subset are labeled in a specific manner.

Another method useful for DC analysis in human lungs is flow cytometry. This method allows accurate identification of DCs by using multiple cell-specific markers and also provides a powerful means of generating quantitative analysis of the frequency of DCs. A major limitation, however, is that this method requires relatively large sized tissues due to the scarcity of DCs in the lungs. Although not common, lung transplantation is sometimes performed for patients with severe interstitial lung diseases (ILDs) [20]. Resected lungs from these patients would be very useful sources for flow cytometric analysis of DCs, which may reveal potential association of DCs with these specific diseases.

In this study, we examined by flow cytometry the prevalence of BDCA1^+^ DCs in the lungs isolated from patients with ILDs: idiopathic pulmonary fibrosis (IPF), hypersensitivity pneumonitis (HP), and chronic obstructive pulmonary disease (COPD). We also identified a new marker for BDCA1^+^ lung DCs, developed a dual-fluorescence staining strategy, and quantified the DCs in bronchial biopsies from patients with asthma [21].

## Materials and Methods

### Ethics

All studies were approved by the UCSF Committee on Human Research, and written informed consent was obtained from all subjects. The interstitial lung disease study was approved by the UCSF IRB #10-00198. For the asthma study, subjects also provided consent for their biospecimens to be placed in the UCSF Airway Tissue Bank IRB #11-05176 for studies. Blood samples were collected as part of a larger study approved by the UCSF IRB #10-02596.

### Interstitial lung disease (ILD) study population

The study population consists of 9 patients with idiopathic pulmonary fibrosis (IPF), 7 with chronic hypersensitivity pneumonitis (HP), 7 with COPD/emphysema, and 13 without lung disease who had died from a non-pulmonary cause. All ILD diagnoses were established thorough multidisciplinary review of clinical data, radiology, and pathology. Diseased lung tissues were obtained from donors undergoing lung transplantation. Normal lung tissues were from lungs originally designated for transplantation by the Northern California Transplant Donor Network, but ultimately not used for various reasons.

### Asthma study population

Asthmatic subjects had been characterized by a prior physician diagnosis of asthma, airway hyper-responsiveness (methacholine PC20<8.0 mg/mL), and at least one of the following: asthma symptoms on 2 or more days per week; β-agonist use on 2 or more days per week; FEV1 less than 85% predicted. Subjects had not taken inhaled or oral corticosteroids for 4 weeks before enrollment. Healthy control subjects had reported no lifetime history of pulmonary disease and lacked airway hyper-responsiveness. Criteria for exclusion for both healthy and asthmatic subjects included a demonstration of any previous history of lung disease other than asthma, a history of an upper or lower respiratory tract infection in the 4 weeks preceding the study enrollment visit, females who were pregnant or breast feeding, taking beta-blocker medication, current cigarette smoking or a total smoking history >15 pack-years. Characterization of Th2-low and -high asthma was performed by microarray hierarchical clustering analysis on epithelial brushings as described previously [21].

### Bronchial biopsy preparation

Endobronchial biopsy samples from healthy and steroid naïve asthmatic subjects were obtained from the Airway Tissue Bank at the University of California, San Francisco. These samples had been collected during multiple research studies performed at UCSF between 2007 and 2013 in which all characterization studies and biospecimen collection had followed standardized protocols. For immunofluorescence microscopy study, biopsies were collected at the lateral edges of 2^nd^ to 5^th^ -order carinae, washed in PBS, fixed in ice-cold 10% formalin, and suspended in paraffin wax. Tissue blocks were arranged with 2–6 biopsies per paraffin block and were cut at a thickness of 3 µm. For flow cytometric analysis, biopsies were not fixed but instead digested with 1 mg/mL collagenase I (Roche) in RPMI without phenol red for 2 hours at 37 degrees, then dispersed with a syringe and passed through a cell filter. The resulting single-cell suspension was spun and resuspended in FACS buffer for staining.

### Peripheral blood mononuclear cell (PBMC) isolation

Blood was collected from healthy anonymous individuals with written and informed consent. PBMCs were isolated by layering blood diluted 1∶1 with HBSS over a Ficoll (GE Sciences) density gradient and subsequently centrifuging it.

### Pulmonary mononuclear cell (PMC) isolation

Lungs were removed from the patient, and large vessels were perfused with PBS. The parenchyma was cut into 2 cm^3^ to 5 cm^3^ sections and placed in cold media for storage until use. Lung specimens were used within 72 hours of isolation. To prepare single cell suspensions for flow cytometry, first the lung specimen was minced with scissors in warm digestion media (20 mL HBSS with 10 mg Collagenase D [Roche] and 2 mg DNAse I [Roche]). Minced tissue was rocked at 37 degrees for 45 minutes. Then the tissue was pushed through a 75 µm-mesh cell strainer over a 50 mL falcon tube with a plunger. The resulting single-cell suspension was spun at 1300 rpm for 5 minutes and washed with HBSS, resuspended in 20 mL HBSS, and layered over 15 mL of Ficoll and spun at 2400 rpm for 20 minutes at room temperature with no brake. Red blood cells and platelets were discarded and the mononuclear cell layer was collected for FACS staining. The resulting cell population was washed once with FACS buffer before staining.

### Flow cytometry

Samples were resuspended in FACS buffer (2% FBS, 0.1% sodium azide) and stained with the following mixtures of Biolegend antibodies: BDCA1-Percp/Cy5.5, CD14-APC, HLADR-Pacific Blue, CD3/19/56-FITC, CD123-PE/Cy7, and either FcεRIα-PE or mouse IgG_2b_-PE, all at manufacturer's recommended concentrations. Some samples were stained with CD45-A700 to identify hematopoietic cells and CD203c-biotin (Biolegend, at manufacturer's recommended concentrations) followed by streptavidin-A647 (Invitrogen, at manufacturer's recommended concentration) to identify mast cells. Cells were stained with antibodies and propidium iodide (PI) (Biolegend) at 1∶400 for 15 minutes at 4 degrees, washed and spun at 1300 rpm, and resuspended in FACS buffer. At least 1×10^6^ cells were run on slow or medium speed on a BD LSRII machine and analyzed with Flowjo software.

### Confocal microscopy

Tissue sections mounted on glass slides were rehydrated in 2 xylene baths for 5 minutes each, 2 100% Ethanol baths for 3 minutes each, and 1 bath of 95% ethanol and 1 bath of 80% ethanol for 1 minute each. Afterwards, slides were rinsed with distilled water and dried. Samples were washed once with 10% goat serum (MP Biomedicals) with 0.05% saponin in PBS (‘wash buffer’) and then stained with 1∶10 human IgG block for 1 hour. Slides were then washed 3 times with wash buffer for 5 minutes each and then stained for 1 hour with 1∶50 anti-FcεRI (CRA-1, Biolegend) and 1∶200 anti-MHCII (“DRAB” polyclonal rabbit antisera). After 1 hour, slides were washed again and stained with 1∶300 goat anti-mouse Alexa 568 and goat anti-rabbit Alexa 488 (Life Technologies) for 1 hour. After rinsing with wash buffer, slides were washed 3 times with PBS and 2 times with distilled water. Slides were dried thoroughly and mounted with Prolong Gold anti-fade mounting reagent with DAPI (Life Technologies). They were then left in the dark at room temperature overnight to cure and were stored at −20 degree for imaging. Slides were imaged using a Nikon C1si confocal microscope. First, overlapping images were taken at 20x and epithelial regions were identified using FIJI software. We calculated the area in microns of the epithelium for each biopsy for each patient. Once regions of epithelium were identified, slides were imaged at 60x to identify FcεRI^+^, MHCII^+^ DCs within the noted regions of epithelium. The numbers of double-positive cells and single-positive FcεRI^+^ cells were counted and recorded for each area of epithelium, and the number of FcεRI^+^ DCs per mm^2^ was quantified for each donor's biopsies. Significance was determined via unpaired, two-tailed Student's T-test. The operator performing the microscopy experiments was blind to clinical status of donor who had provided the tissue section.

## Results

### BDCA1^+^ DCs markedly increase in the lungs of patients with IPF or HP

**Figure 1:**
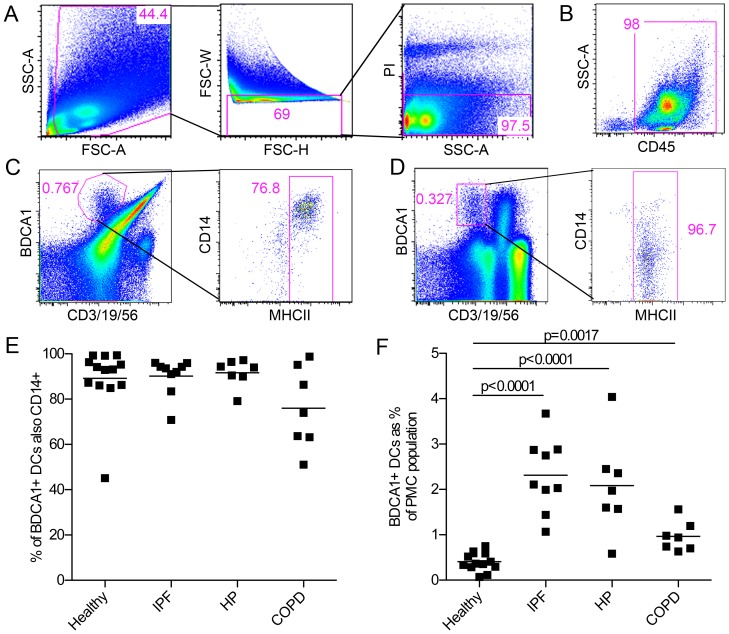
BDCA1^+^ DCs markedly increase in the lungs of IPF and HP. (**A**) Pulmonary mononuclear cells were isolated from surgically resected parenchymal lung tissue. Live singlet cells were identified based on forward and side scatter and a lack of PI-staining. Data from one representative donor of over 20 are shown. Numbers in pink represent the gate population as a % of parent. (**B**) Live, singlet cells were stained with an anti-CD45 antibody to determine the prevalence of hematopoietic cells. Data from one representative donor of 3 healthy and 6 ILD patients are shown. (**C**) BDCA1^+^ DCs were identified in PMC isolates by their expression of BDCA1, MHCII, and their lack of expression of the lymphocyte markers CD3, CD19, and CD56. Data from one representative donor of over 20 are shown. (**D**) DCs were identified from peripheral blood mononuclear cells in a similar fashion based on their expression of BDCA1, and their lack of expression of the lymphocyte markers CD3, CD19, and CD56. They were further examined for their expression of MHCII. Data from one representative donor of over 20 are shown. (**E**) Percentage of BDCA1^+^ lung DCs that are also CD14^+^ from a cohort of donors described in Table 1. Each dot represents a unique donor and bar represents mean. There are no significant differences between subject groups as measured by unpaired, two-tailed Student's t-test. (**F**) BDCA1^+^ DCs were determined as a percentage of total PMCs from the same cohort of donors. Each dot represents a unique donor and bar represents mean. Statistical significance was determined by unpaired, two-tailed Student's t-tests.

To examine whether DCs infiltrate the lungs in association with human interstitial lung diseases, we isolated pulmonary mononuclear cells (PMCs) from lung parenchyma of healthy deceased donors and patients with IPF, HP, and COPD (Table 1) and identified DCs by flow cytometry. First, singlet, live cells were gated based on forward and side scatter and a lack of PI staining (Fig. 1A). A great majority of these cells, even in fibrotic lungs, were found to be of hematopoietic origin as they were CD45^+^ (Fig. 1B). Next, BDCA1^+^ DCs were identified by gating BDCA1^+^lymphocyte marker^-^ cells and further gating MHCII^+^ cells (Fig. 1C). In contrast to blood BDCA1^+^ DCs (Fig. 1D), we found that lung BDCA1^+^ DCs were mostly CD14^+^ for all donors tested (Fig. 1E). A quantitative analysis of DC frequencies revealed that BDCA1^+^ DCs represented around 0.1-0.9% of PMCs in healthy lungs (Fig. 1F) and that this frequency increased in both of IPF and HP patients by five to six fold (Fig. 1F). COPD patients also had a significant increase in BDCA1^+^ DCs but to a much lesser degree than patients with fibrotic diseases (Fig. 1F). Thus, IPF and HP are associated with a robust increase in BDCA1^+^ DCs while COPD is associated with relatively a modest increase.

**Table 1:** Subject data for ILD cohort.

		IPF	HP	COPD/Emphysema
**Number**		9	7	7
**Age ± SD**		60±7	52±14	62±7
**Gender M:F (% F)**		5∶4 (44%)	3∶4 (57%)	4∶3 (43%)
**Ethnicity**	White	7	5	5
	African American	0	0	2
	Asian	0	0	0
	Pacific Islander	0	0	0
	Hispanic	2	2	0
**FEV % predicted ± SD**		59±17	40±10	18±6

Healthy discarded donor lungs or diseased lungs from transplantation were examined for DC content of pulmonary mononuclear cells (PMCs). All ILD diagnoses were made using accepted standard diagnostic criteria. Lungs were lavaged and stored at 4 degrees in media until use (<72 hours from harvest). Age and FEV % predicted are shown as mean ± SD.

### BDCA1^+^ DCs express FcεRI in lung parenchyma and airways

To aid in identification of BDCA1^+^ DCs in microscopic studies using small lung biopsies, we searched for specific markers that could be used to better identify these cells. Previous studies have shown that the high affinity IgE receptor, FcεRI, is expressed not only in mast cells and basophils but also in BDCA1^+^ DCs of human tissues. Blood and tonsil BDCA1^+^ DCs and Langerhans cells, the skin epidermal DC subset, express FcεRI [22]–[24]. BDCA1^+^ DCs from arthritic synovial fluid and malignant tumor ascites also express FcεRI [15]. Whether lung BDCA1^+^ DCs express FcεRI has not been examined, although CD1a^+^ DCs in the airways have been shown to express FcεRI by immunohistochemistry [25].

**Figure 2:**
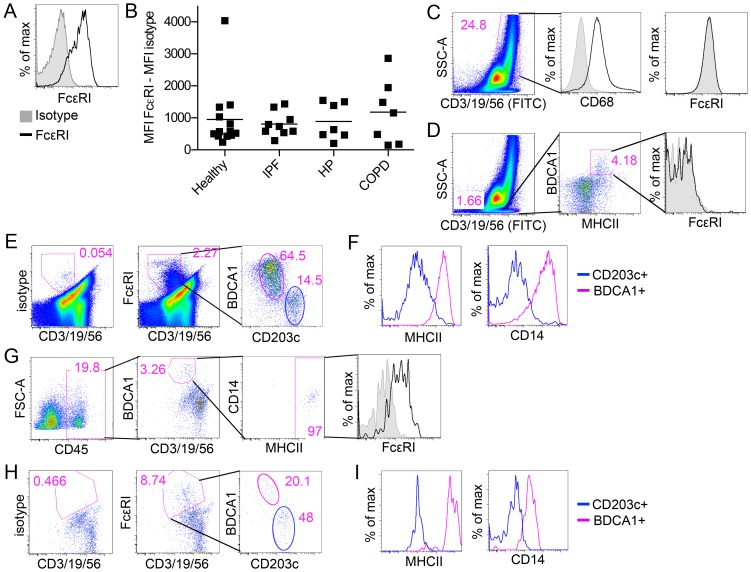
Lung BDCA1^+^ DCs express FcεRI. (**A**) Lung BDCA1^+^ DCs from one representative donor were gated using the strategy as described in Figure 1 and examined for expression of FcεRI. Isotype control is shown in grey and anti-FcεRI antibody is in black. (**B**) Mean fluorescence intensity of FcεRI signal minus isotype control intensity in lung BDCA1^+^ DCs was plotted for each donor tested in Figure 1. Bar represents mean. There are no significant differences between subject groups as measured by unpaired, two-tailed Student's t-test. (**C**) Macrophages were gated based on high autofluorescence reflected to the FITC channel and high side scatter and tested for their expression of CD68 and FcεRI. Isotype control is shown in grey and anti-CD68 antibody or FcεRI antibody is in black. (**D**) B-cells were gated based on lymphocyte marker (CD3/19/56)^+^SSC^lo^MHCII^+^BDCA1^+^ cells and tested for their expression of FcεRI. Isotype control is shown in grey and anti-FcεRI antibody is in black. (**E**) FcεRI^+^ cells from lung parenchyma were identified by comparing isotype control antibody-stained PMCs (left panel) to FcεRI antibody-stained PMCs (middle panel). FcεRI^+^ cells from the middle panel were examined for the expression of the DC marker BDCA1 and the mast cell/basophil marker CD203c (right panel). (**F**) Cell populations gated in Fig. 2E (right panel) were compared for expression of MHCII and CD14; only BDCA1^+^ cells (pink line) are MHCII^+^CD14^+^. (G) BDCA1^+^ DCs were identified from airway biopsies and were examined for FcεRI expression. Among CD45^+^ cells, BDCA1^+^ DCs were gated as described in Fig. 1C and examined for the expression of FcεRI^+^. Isotype control is shown in grey and anti-FcεRI antibody is in black. (H) FcεRI^+^ cells from airway biopsies were identified and analyzed for the expression of BDCA1 and CD203 as described in Fig. 2E. (I) Cell populations gated in Fig. 2H (right panel) were compared for expression of MHCII and CD14; only BDCA1^+^cells (pink line) are MHCII^+^CD14^+^.

First, we examined FcεRI expression in human lung BDCA1^+^ DCs by flow cytometry. We found that lung BDCA1^+^ DCs bound an anti-FcεRI antibody significantly more than an isotype control antibody, indicating that these cells indeed express FcεRI (Fig. 2A). Next, we examined how stably FcεRI is expressed in BDCA1^+^ DCs using our cohort of healthy and diseased lungs. We found that FcεRI was expressed in BDCA1^+^ lung DCs throughout cohort to varying degrees (Fig. 2B), which is consistent with previous reports of varying FcεRI levels in BDCA1^+^ DCs in the blood [22], [26], [27]. Despite this variation, surface FcεRI was detectable for all donors examined (Fig. 2B), and the average expression levels did not significantly differ between health and disease (Fig. 2B). This finding indicates that FcεRI is stably expressed in BDCA1^+^ DCs in the lungs.

We also examined whether FcεRI was expressed in cell types other than BDCA1^+^ DCs in the lungs. First, macrophages were examined. These cells were gated by high autofluorescence and high side scatter (Fig. 2C). This gating strategy has been previously used by others [13], and further validated by us confirming the expression of human macrophage markers such as CD68 in gated cells (Fig. 2C). No FcεRI expression was detected in these cells (Fig. 2C, right panel). Secondly, B-cells were examined. They were identified as [CD3/19/56 antibody mixtures]^+^MHCII^+^ BDCA1^+^ (Fig. 2D). No FcεRI expression was detected in these cells, either (Fig. 2D, right panel). Lastly, to get a more comprehensive view of FcεRI-expressing cells present in the lungs, we gated all PMCs that were non-autofluorescent but labeled by FcεRI antibody (Fig. 2E), and determined which cells are included in this gating. Expression of CD203c, a specific marker of human mast cells and basophils, and BDCA1 were examined. We found that FcεRI^+^ PMCs were segregated into two distinct populations that were either CD203^+^ or BDCA1^+^ (Fig. 2E, right panel). This finding suggested to us that the FcεRI^+^ PMCs are almost entirely composed of basophils/mast cells and BDCA1^+^ DCs. Further examination of the BDCA1^+^ cells showed that they were MHCII^+^CD14^+^ (Fig. 2F), confirming they were indeed BDCA1^+^ DCs. Conversely, CD203c^+^ cells had very low levels of MHCII and CD14 (Fig. 2F). This finding indicates that mast cells/basophils and BDCA1^+^ DCs are the only significant cell types expressing FcεRI in human lungs.

To expand our examination of FcεRI expression in BDCA1^+^ DCs in lung parenchyma, we also examined BDCA1^+^ DCs in airways. Two airway bronchial biopsies were obtained from a patient with asthma and the whole biopsy digests, rather than PMCs, were analyzed by flow cytometry to maximize the recovery of cells. By looking only at CD45^+^ cells, we found a distinct population of cells that express BDCA1 but not lymphocyte markers (Fig. 2G). A majority of these cells were MHCII^+^ (Fig. 2G), confirming that they are BDCA1^+^DCs. Furthermore, we found that these DCs expressed FcεRI similar to what we found with parenchymal DCs (Fig. 2G, right panel). We also analyzed the composition of FcεRI^+^ cells in the airway by taking the same approach described above for parenchymal tissues. We found that like in the parenchyma, FcεRI^+^ cells in the airways were segregated into either BDCA1^+^ or CD203c^+^ cells (Fig. 2H), and that BDCA1^+^ cells but not CD203^+^ cells expressed MHCII and CD14 (Fig. 2I). Thus, mast cells/basophils and BDCA1^+^ DCs are the only cell types expressing FcεRI in the airways. These findings indicate that FcεRI can be used as a marker of BDCA1^+^ DCs in human lungs and airways when combined with either BDCA1, MHCII, or CD14.

### BDCA1^+^ DCs increase in the epithelium of Th2-high but not Th2-low asthmatic airways

Having found that BDCA1^+^ DCs in human airways can be identified by their co-expression of FcεRI and MHCII, we examined the prevalence of BDCA1^+^ DCs in asthma by employing immunofluorescence microscopy of bronchial biopsies using FcεRI and MHCII antibodies. Recent studies have illustrated that human asthma is heterogeneous and can be segregated into two distinct subsets based on differential expression of Th2-responsive genes in the airway epithelium [21]. “Th2-high” asthma is characterized by high levels of Th2-responsive gene expression and it is associated with mast cell infiltration, sub-epithelial fibrosis, and eosinophilia [21]. In comparison, “Th2-low” asthma is characterized by the usual airflow dysfunction but relative absence of mast cell infiltration, sub-epithelial fibrosis, and eosinophilia [21].

**Figure 3:**
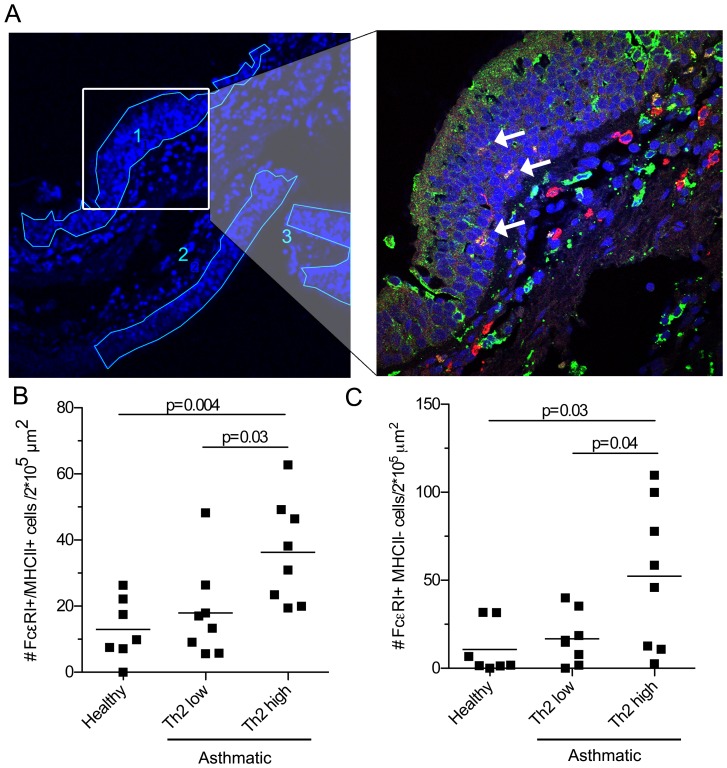
BDCA1^+^ DCs increase in the epithelium of Th2-high but not Th2-low asthmatic airways. (**A**) Left panel, representative image of bronchial airway biopsy showing DAPI staining in blue (other channels are omitted for clarity). Lumen is located at the image peripheries and sub-epithelial tissues are centrally located. Images were obtained by a Nikon C1si microscope at 20x magnification. Areas of airway epithelium (numbered) were manually outlined using FIJI software and area was calculated for each individual biopsy. Region highlighted in left was imaged at 60x as shown in right panel (lumen is located to the top left); DCs were identified based on expression of MHCII (green) and FcεRI (red) and presence of significant cell body. Double-positive DCs in epithelium are indicated with arrows. (**B–C**) FcεRI^+^ MHCII^+^ double positive cells (B) and FcεRI^+^ single positive cells (C) in the epithelium were counted for each biopsy. Number of cells per 2×10^5^ µm^2^ epithelium was calculated. Bar represents mean. Statistical significance (p values) was determined via two-tailed, unpaired Student's t-test.

We examined bronchial biopsies of eight Th2-low asthmatics and eight Th2-high asthmatics compared to seven healthy controls (Table 2). 3 µm-thick biopsy sections were stained with FcεRI and MHCII antibodies and subsequently with fluorophore-conjugated secondary antibodies. Images of each section were taken by confocal microscopy at 20x magnification and the area of epithelium was manually marked with Fiji software (Fig. 3A, left panel), which was then quantified as µm^2^/biopsy. The same section was then imaged at 60x and the number of FcεRI^+^MHCII^+^ cells in the epithelium was counted. (Fig. 3A, right panel). The reason that we analyzed DCs only within the epithelium is that Th2-low and -high asthma had been characterized based on gene expression profiles of the airway epithelium [28]. In addition, recent mouse studies suggest a significant contribution of epithelial cell-to-DC crosstalk to asthma pathology [29], implicating the important role of epithelial DCs in this disease. We found that the number of FcεRI^+^MHCII^+^ cells was significantly increased in the epithelium of Th2-high asthmatics compared to Th2-low asthmatics and healthy controls (Fig. 3B). Thus, BDCA1^+^ DCs accumulate in the airway epithelium of Th2-high asthma but not Th2-low. We also counted the number of epithelial FcεRI^+^MHCII^-^ cells - presumably mast cells or basophils - and found that these cells also significantly increased in Th2-high asthmatics but not in Th2-low asthmatics (Fig. 3C), consistent with previous findings [30].

**Table 2:** Subject data for asthmatic cohort.

		Healthy	Asthmatic	
			*Th2-low*	*Th2-high*
**Number**		7	8	8
**Age ± SD**		36±12	30±12	41±8
**Gender M:F (% F)**		5∶2 (29%)	6∶2 (25%)	5∶3 (38%)
**Ethnicity**	White	5	5	6
	African American	0	1	1
	Asian	1	1	1
	Pacific Islander	0	0	0
	Hispanic	1	1	0
**FEV1 % predicted ± SD**		92%±7	91%±10	79%±10

Healthy or asthmatic subjects were enrolled and tested for airway hyper-responsiveness as measured by airway hyper-responsiveness upon methacholine challenge and allergies as measured by serum IgE. Th2 skew was categorized by methods from Woodruff et al. [21]. Age and FEV % predicted are shown as mean ± SD.

## Discussion

Our study demonstrates that IPF is associated with a robust (5–6 fold) increase in BDCA1^+^ DCs in the parenchyma. By quantifying DCs as a percentage of PMCs, we show a specific increase in DCs as opposed to overall increase in hematopoietic cells. Others have shown a significant increase in DCs in bronchoalveolar lavage from IPF patients [31], consistent with our finding. We also found that HP is associated with a dramatic increase in BDCA1^+^ DCs in lung parenchyma. To our knowledge, this is the first study that examined DC frequency in HP in a quantitative manner. We also found a significant increase in the frequency of DCs in the lungs of COPD, but to a much lesser degree (∼2 fold) than IPF or HP. Others have examined the frequency of the same interstitial lung DC subsets in COPD and found a slight increase (up to ∼2 fold) in the median DC frequency compared to healthy controls [32], although it did not reach significance in their statistical analysis. Notably, this study had significant patient variation and used a different method of statistical analysis, which may have lowered their threshold for statistical significance.

Our finding that DCs dramatically increased in IPF and HP implicates a potential contribution of DCs to these two diseases. As DCs are potent activators of T cells, they may contribute to lymphocyte-mediated inflammation in these diseases. Consistent with this hypothesis, chemokines that favor recruitment and accumulation of DCs and lymphocytes have been shown to significantly increase in the bronchoalveolar lavage fluid and lungs of IPF or HP patients [7], [33], [34]. While there are notable infiltrates of mixed lymphocytic populations in IPF lungs [35], the major pathology associated with IPF is progressive, extensive fibrosis of the lungs [36], and the ineffectiveness of immunosuppressant treatment such as corticosteroids [37] has led to the belief that immune cells are not likely involved in the disease process. Like IPF, late stage, chronic HP is also associated with severe fibrosis in the lungs [38], though there is also a significant contribution of lymphocytic inflammation especially at early stages of the disease [39]. Alternatively, or in addition to their role in activating inflammatory T cells, DCs may significantly contribute to fibrosis in these diseases. In a mouse model of bleomycin-induced lung fibrosis, pharmacological inactivation of DCs resulted in a marked reduction in fibrosis [40]. Furthermore, DCs in IPF accumulate in fibrotic foci [7] and recent evidence indicates that they can regulate the vasculature and function of fibroblast-type cells [41]. Indeed, DCs can express TGF-β1, which could potentially act directly on mesenchymal cells to modulate their behavior to be more profibrotic [42], [43].

Our studies indicate that FcεRI can serve as a useful marker of BDCA1^+^ lung DCs. FcεRI expression by antigen presenting cells in human lungs has been previously examined by immunohistochemistry, which showed that some CD1a^+^ cells were FcεRI^+^ [25]. However, the extent to which FcεRI is expressed in a more comprehensive lung DC subset has not been examined. Our study indicates that FcεRI is constitutively expressed in BDCA1^+^ lung DCs, similarly to what had been found in blood, tonsil [22], and intestine [44]. Furthermore, a recent study revealed that FcεRI is expressed in BDCA1^+^ DCs in human arthritic synovial fluid and malignant tumor ascites [15], and additionally demonstrated that FcεRI is a useful marker that distinguishes DCs from macrophages, which do not express FcεRI but do express mannose receptors and DC-SIGN [15]. Collectively, these and our findings suggest that FcεRI can serve as a useful marker for BDCA1^+^ DCs in the lungs and possibly many other human tissues.

We found that the majority of BDCA1^+^ DCs in the lungs express CD14 while those in the blood do not. A previous study has shown that BDCA1^+^ DCs in tissues of inflammatory conditions express CD14 and suggested that CD14 expression is a specific feature of inflammatory DCs [15]. However, this study did not analyze DCs from any non-inflammatory tissue environment. Therefore, it was unclear whether CD14 expression is linked to the inflammatory environment or due to the distinct property of tissue DCs versus blood DCs. When we examined BDCA1^+^ DCs in healthy lungs, we found that most express CD14. Thus, we suggest that CD14 expression is not a marker of inflammatory DCs, but a marker of tissue DCs as opposed to circulating blood DCs. Interestingly, a minor fraction of BDCA1^+^ DCs in the blood also express CD14 along with FcεRI; these cells may represent DCs that are initiating or are in the process of migrating into peripheral tissue sites.

By employing dual-antibody immunofluorescence microscopy using FcεRI and MHCII antibodies, we found that BDCA1^+^ DCs are enriched specifically in Th2-high asthmatic airway epithelium. One might suspect that DCs of healthy and Th2-low asthmatics were undercounted in association with their FcεRI expression levels compared to Th2-high asthmatics, as Th2-associated conditions such as high serum IgE tend to correlate with surface FcεRI expression [27]. However, we consider this unlikely because we were able to readily localize rare FcεRI^low^ cells by using a high laser power and a high magnification optic (60X), and thus were able to clearly distinguish them from FcεRI^-^ cells. One might also argue that basophils or mast cells contaminated our DC identification strategy because of potential MHCII expression in these cells. Besides remaining controversy surrounding MHCII expression levels in lung mast cells or basophils [45], [46], we found their MHCII expression levels to be 10-100-fold lower than DCs (Fig. 2F), even in Th2-high asthmatic airways (Fig. 2I). This large difference was readily distinguishable with fluorescence microscopy by keeping the laser power within a non-saturating range of MHCII levels. Thus, it is unlikely that mast cells or basophils were included in our MHCII^+^FcεRI^+^ cell counting.

Our finding of BDCA1^+^ DC accumulation in Th2-high asthma is consistent with data in mouse models of asthma, and the functional role of BDCA1^+^ DCs in asthma can be predicted from these studies. Depletion of mouse lung DCs or inhibition of their function has been shown to abrogate asthma-like pathology in mouse models [5], [47]. DCs also have been found to play a critical role in inducing and maintaining Th2 immunity in mouse lungs [47], [48]. Similar to DCs in mice, BDCA1^+^ DCs in humans may play a crucial role in generating and maintaining Th2 immunity in asthma. Notably, Th2-high asthma typically responds well to steroid therapy [21], and steroid treatment has been shown to lower the number of DCs in airways [49]. DC accumulation may be tightly connected to disease exacerbation; however, the specific mechanism by which airway epithelial DCs accumulate remains to be determined. Epithelial cells in Th2-high asthma express a number of cytokines and chemokines [28] which may recruit DCs to the epithelium. Alternatively, DCs may proliferate in the epithelium of Th2-high asthmatics; interestingly, a recent study has shown that airway epithelial DCs but not interstitial DCs proliferate in a Th2 mouse asthma model [50]. Given the central role of DCs in inducing and perpetuating Th2 inflammation in mouse and possibly human asthma, understanding the mechanism by which DCs increase in the airway epithelium may provide a novel therapeutic approach to control this disease.

In conclusion, by using flow cytometry and dual-color immunofluorescence microscopy we found that IPF, HP, COPD, and Th2-high asthma are associated with the accumulation of BDCA1^+^ DCs at the sites of disease pathology. Further biochemical and functional analyses of this DC subset are expected to improve our understanding of the pathogenesis of these diseases.
